# The role of adolescent social relationships in promoting alcohol resistance: Interrupting the intergenerational transmission of alcohol misuse

**DOI:** 10.1017/s0954579422000785

**Published:** 2022-08-12

**Authors:** Mallory Stephenson, Fazil Aliev, Sally I-Chun Kuo, Alexis C. Edwards, Gayathri Pandey, Jinni Su, Chella Kamarajan, Danielle Dick, Jessica E. Salvatore

**Affiliations:** 1 Virginia Institute for Psychiatric and Behavioral Genetics, Virginia Commonwealth University, Richmond, VA, USA; 2 Department of Psychology, Virginia Commonwealth University, Richmond, VA, USA; 3 Department of Psychiatry, Robert Wood Johnson Medical School, Rutgers University, New Brunswick, NJ, USA; 4 Department of Psychiatry, School of Medicine, Virginia Commonwealth University, Richmond, VA, USA; 5 Department of Psychiatry and Behavioral Sciences, Downstate Medical Center, State University of New York, Brooklyn, NY, USA; 6 Department of Psychology, Arizona State University, Tempe, AZ, USA

**Keywords:** adolescence, alcohol, parenting, peer relationships, resistance

## Abstract

Genetic factors contribute to the intergenerational transmission of alcohol misuse, but not all individuals at high genetic risk develop problems. The present study examined adolescent relationships with parents, peers, and romantic partners as predictors of realized resistance, defined as high biological risk for disorder combined with a healthy outcome, to alcohol initiation, heavy episodic drinking, and alcohol use disorder (AUD). Data were from the Collaborative Study on the Genetics of Alcoholism (*N* = 1,858; 49.9% female; mean age at baseline = 13.91 years). Genetic risk, indexed using family history density and polygenic risk scores for alcohol problems and AUD, was used to define alcohol resistance. Adolescent predictors included parent-child relationship quality, parental monitoring, peer drinking, romantic partner drinking, and social competence. There was little support for the hypothesis that social relationship factors would promote alcohol resistance, with the exception that higher father-child relationship quality was associated with higher resistance to alcohol initiation ($\hat{\beta }=-0.19$, 95% CI = −0.35, −0.03). Unexpectedly, social competence was associated with *lower resistance* to heavy episodic drinking ($\hat{\beta }=0.10$, 95% CI = 0.01, 0.20). This pattern of largely null effects underscores how little is known about resistance processes among those at high genetic risk for AUD.

Family history and genetic loading for alcohol use disorder (AUD) are well-established risk factors for the development of alcohol use and problems among adolescents and young adults (Chassin et al., 2004; Verhulst et al., 2015). Parental AUD is associated with early initiation of alcohol use, elevated risk for binge drinking in adolescence, and more rapid progression from alcohol use initiation to AUD diagnosis (Chassin et al., 2002, 2004; Hussong & Bauer, 2008). Twin and adoption studies have explored the heritability of alcohol problems, demonstrating that approximately 50% of the variation in AUD is attributable to genetic influences (Verhulst et al., 2015). Further, molecular genetic studies suggest that AUD is highly polygenic, driven by hundreds or thousands of genetic variants of very small individual effect (Sullivan et al., 2012).

Nonetheless, many individuals with high familial or polygenic loading do not develop alcohol problems and may be considered “resistant” to AUD. The substance use literature has traditionally adopted a risk perspective, identifying environmental factors that increase risk for alcohol use and misuse. As a result, the mechanisms through which individuals at high familial or genetic risk may develop resistance to AUD remain poorly understood. Vanyukov et al. (2016) identify several targets to further understand alcohol resistance: (1) outset resistance, defined as low biological and environmental risk from birth; (2) realized resistance, described as high biological risk or childhood behavioral risk for a disorder combined with a healthy adult outcome; (3) resistance to continued drug use after initiation; (4) resistance to addiction; (5) ability to recover after becoming addicted; and (6) resistance to relapse after recovery.

Previous studies have focused on addiction resistance (Hoffmeister et al., 2019; Kendler & Myers, 2015), the fourth facet of the alcohol resistance phenotype. Individuals with high levels of addiction resistance report fewer substance use disorder (SUD) symptoms than would be expected based on their maximal drug consumption. Conversely, individuals with low levels of addiction resistance report more SUD symptoms than expected. In one study, Kendler and Myers (2015) found that parental history of alcohol or drug use disorders, low parental warmth, and co-morbid internalizing and externalizing disorders predicted lower levels of addiction resistance. Similarly, Hoffmeister et al. (2019) identified behavioral disinhibition, poor emotional stability, and early life adversity as negative predictors of addiction resistance (Hoffmeister et al., 2019).

In contrast with addiction resistance, the remaining facets of alcohol resistance are relatively unexplored. Given that approximately 7.5 million children in the United States live with at least one parent affected by AUD (Lipari & Van Horn, 2017), efforts to characterize the development of realized resistance, defined as lower levels of substance use or fewer SUD symptoms than would be expected based on one’s biological or childhood behavioral risk for disorder (Vanyukov et al., 2016), have the opportunity to benefit a substantial proportion of the population and provide insight into potential targets for the prevention of alcohol use and problems. In the present study, we adopt an organizational-developmental perspective to understand realized resistance. Specifically, we evaluate the contributions of adolescent social relationships with parents, peers, and romantic partners to delayed onset of alcohol use, later initiation or absence of heavy episodic drinking, and a lack of AUD diagnosis among individuals with high genetic risk (Vanyukov et al., 2016).

## The organizational-developmental model

An organizational-developmental perspective posits that development is defined by changes in the organization of behavior over time. Within each developmental period, behavior must shift to accommodate salient developmental issues (Sroufe et al., 2005), which are guided by sociocultural and historically embedded expectations for successful adaptation (Roisman et al., 2004). During adolescence in a Western context, the primary developmental task is individuation, or cultivating a sense of being a unique person. This maturing awareness of identity is facilitated by successfully balancing autonomy with connectedness, developing peer network competence, and coordinating school, work, and social activities (Sroufe et al., 2005).

Early adolescence signifies a critical shift in the parent-child relationship, as parents must monitor and remain close to their child while also promoting their expanding autonomy. Adolescents increasingly develop social competence, drawing on experiences with their parents to approach relationships with a complex network of same- and other-gender friends and romantic partners (Sroufe et al., 2005). Therefore, the organizational-developmental model posits that features of the parent-child relationship facilitate the development of social competence and inform adolescents’ relationships with their peers and romantic partners, which in turn contribute to healthy (or unhealthy) adaptation and functioning. If we consider alcohol resistance to be a healthy developmental outcome, the organizational-developmental model implicates two non-mutually exclusive pathways to alcohol resistance in adolescence. First, features of the parent-child relationship, including relationship quality and parental monitoring, may influence whether adolescents select into higher versus lower drinking peer groups and romantic partnerships, affecting alcohol resistance. Second, positive parent-child relationship characteristics may bolster adolescent social competence and enhance alcohol resistance. We review the potential contributions of parenting, alcohol use among peers and romantic partners, and social competence in turn. In view of the limited work on alcohol resistance, we focus on prior studies of the associations between adolescent social relationship factors and alcohol-related outcomes more broadly.

## Parenting and adolescent drinking behavior

Parental monitoring and parent-child relationship quality have well-established protective effects on alcohol use onset, levels of alcohol use, and initiation of binge drinking (Handren et al., 2016; Rusby et al., 2018; Ryan et al., 2010; Van Ryzin et al., 2012). For example, Yap et al. (2017) explored the joint and separate effects of parenting by mothers and fathers on adolescent alcohol use in a systematic review and meta-analysis. When they examined 12 dimensions of parenting relevant to offspring drinking patterns, parental monitoring emerged as the strongest protective factor, accounting for 3% of the variance in alcohol use initiation and 5% of the variance in subsequent use and misuse. Maternal support was related to delayed alcohol use initiation, and both maternal and paternal support predicted lower alcohol use and misuse. Parental involvement, mother-child relationship quality, and father-child relationship quality similarly predicted later alcohol use onset and lower levels of consumption (Yap et al., 2017).

In addition to effects on alcohol use initiation, prior studies suggest that parental monitoring and parent-child relationship quality reduce risk for heavy episodic drinking and alcohol problems. Parental monitoring is negatively associated with alcohol misuse, binge drinking, and the development of SUDs across adolescence and young adulthood (Barnes et al., 2000; Bountress et al., 2017; Donaldson et al., 2016), and these protective effects are particularly pronounced for those at high genetic risk (Bountress et al., 2017; Dick, Viken, et al., 2007; Salvatore et al., 2014). Similarly, maternal positive parenting is negatively associated with heavy episodic drinking in adolescence (Su et al., 2018), and poor parent-child relationships are related to increased risk for heavy episodic drinking, as well as AUD onset and persistence (Berg et al., 2018; Foster et al., 2014).

## Alcohol use by peers and romantic partners, social competence, and drinking behavior

Affiliations with drinking peers may contribute to lower levels of alcohol resistance. Perceived peer substance use is associated with increased risk for alcohol use initiation (Bucholz et al., 2017), and affiliating with a greater number of friends who drink predicts more rapid increases in alcohol consumption across adolescence (Simons-Morton & Chen, 2005). Substance use by close friends is also related to higher levels of heavy episodic drinking in adolescence, a greater increase in heavy episodic drinking from mid-adolescence to young adulthood (Li et al., 2017), and higher likelihood of meeting criteria for substance abuse or dependence prior to age 21 (Jones et al., 2016). Moreover, studies of gene-environment interaction suggest that genetic influences on alcohol use increase with increases in friends’ alcohol use, suggesting that peer drinking may be particularly detrimental to the development of realized resistance (Dick, Pagan, et al., 2007; Harden et al., 2008).

Fewer studies have evaluated connections between adolescent romantic relationships and drinking behavior. Yet, romantic relationships are an emerging developmental task in adolescence (Roisman et al., 2004) and may have particular significance for the development of alcohol resistance. Romantic relationships share many features with friendships in adolescence (Furman & Shaffer, 2003; Gudonis et al., 2012). However, adolescent romantic relationships rarely emerge from friendships or proximal peer groups, instead facilitating access to a new peer environment (Kreager et al., 2016). Further, adolescent romantic relationships are characterized by greater intensity, increased expressions of affection (Collins et al., 2009), higher support, and greater conflict when compared to peer relationships (Furman & Shomaker, 2008). Due to the emotional intensity and frequency of contact, adolescent romantic relationships may exert behavioral influence in spite of their brevity. Consistent with this idea, drinking by romantic partners has been prospectively associated with adolescent alcohol use, particularly among individuals with initially low levels of drinking (Gudonis et al., 2012). The effects of romantic partners’ drinking may be especially relevant for female adolescents in heterosexual relationships, as relationships with older boyfriends provide increased access to alcohol (Bremner et al., 2011; Brown et al., 2009).

Finally, adolescent social competence, defined by the quality of one’s social interactions and relationships, group status, and social self-efficacy (Rose-Krasnor, 1997), may contribute to the development of resistance to alcohol use onset, initiation of binge drinking, and AUD. The organizational-developmental model (Sroufe et al., 2005) proposes that successful adaptation in adolescence is evidenced by competently navigating a complex network of same-and other-gender friends, as well as romantic partners. Assuming that alcohol resistance can be considered a healthy developmental outcome, the organizational-developmental perspective underscores the potential importance of adolescent social competence in the development of alcohol resistance among individuals with high genetic risk.

## Evaluating pathways to alcohol resistance as a function of race/ethnicity and sex

Though prior research highlights parental monitoring, parent-child relationship quality, drinking by peers and romantic partners, and social competence as potential predictors of alcohol resistance, it is plausible that pathways to alcohol resistance may differ according to adolescents’ racial/ethnic background and sex. Prior research has shown differences in the patterns and correlates of drinking behavior by race/ethnicity (Bersamin et al., 2005; Chartier et al., 2009) and sex/gender (Dir et al., 2017; Foster et al., 2015; Kendler et al., 2015; Salvatore et al., 2017). For example, Black adolescents tend to initiate alcohol use later than White adolescents, are less likely to continue to progress to regular use, and exhibit lower rates of heavy episodic drinking (Malone et al., 2012; Patrick & Schulenberg, 2010). Further, affiliating with friends who get drunk is a stronger predictor of heavy episodic alcohol use among White adolescents when compared to Black adolescents (Patrick & Schulenberg, 2010). Though some studies suggest that the association between parental monitoring and substance use is stronger for White adolescents when compared to Black adolescents (Blustein et al., 2015; Bohnert et al., 2009), a study conducted by Su et al. (2018) found that the effects of parenting behavior on risky drinking were similar among European American and African American individuals. Thus, despite well-established differences in patterns of drinking behavior between White and Black adolescents, potential differences in the pathways to alcohol resistance remain unclear.

Differences in adolescent drinking patterns have also been observed between males and females. Though male and female adolescents report similar rates of alcohol use initiation (Swendsen et al., 2012), males exhibit greater risk for heavy drinking, binge drinking, and alcohol dependence (Chassin et al., 2002; Stone et al., 2012). Further, there is some evidence that family relationships and parenting behaviors are more influential for females when compared to males (Beyers & Seiffge-Krenke, 2007), though many studies have not found evidence for disparate effects of parenting according to child sex. For example, Rusby et al. (2018) found that the effects of parent-child relationship quality and parental monitoring on binge drinking onset were similar for males and females.

## The current study

In the present study, we examined the contributions of adolescent social relationships with parents, peers, and romantic partners to realized alcohol resistance. Realized alcohol resistance was operationalized as delayed alcohol use initiation, later initiation or absence of heavy episodic drinking, and a lack of AUD diagnosis among individuals with high familial or polygenic loading for AUD. Our hypotheses were as follows:

1. Adolescents who report higher quality relationships with their mother and father, greater parental monitoring, and higher levels of social competence will exhibit higher levels of alcohol resistance.
2. Conversely, individuals who report affiliations with drinking peers and romantic partners will demonstrate lower levels of resistance.
3. Consistent with the organizational-developmental model, mother-child relationship quality, father-child relationship quality, and parental monitoring will be positively associated with adolescent social competence, which in turn will predict higher alcohol resistance. In addition, mother-child relationship quality, father-child relationship quality, and parental monitoring will be negatively associated with peer and romantic partner drinking. Fewer affiliations with drinking peers and romantic partners will then predict higher levels of alcohol resistance.

Finally, we conducted exploratory analyses to test whether predictors of alcohol resistance differ as a function of adolescent race/ethnicity (operationalized as ancestry) and sex. The hypotheses and analytic plan for this study were preregistered using the Open Science Framework (https://osf.io/rkbc5).

## Method

### Participants

Participants were 1,858 adolescents and young adults (49.9% female) from the Collaborative Study on the Genetics of Alcoholism (COGA) Prospective Sample (Bucholz et al., 2017), a large-scale longitudinal study of approximately 2,255 families densely affected by alcoholism (Begleiter et al., 1995). Data collection began in 2004 and concluded in 2019, with participants assessed biennially. The current analyses were limited to individuals who completed the adolescent version of the Semi-Structured Assessment for the Genetics of Alcoholism for Children (C-SSAGA-A-IV) during their baseline assessment (Bucholz et al., 1994; Hesselbrock et al., 1999). The self-reported race/ethnicity of participants was 28.5% Black/African American, 49.7% White/Caucasian, and 21.8% Native American, Asian, Pacific Islander, or Other. Participants were an average age of 13.91 years (*SD* = 1.78 years, range = 11–17 years) at baseline and were re-assessed every two years. Approximately 62% of participants completed at least one follow-up assessment, and participants completed a maximum of four assessments (i.e., one baseline assessment and three follow-up assessments). Parenting measures were drawn from the baseline assessment; measures of peer drinking, romantic partner drinking, and social competence were from the first follow-up assessment; and all available assessments were used to derive the alcohol resistance measures.

### Measures

#### Parent-child bonding

Father- and mother-child bonding were assessed using the 12-item care scale of the Parental Bonding Instrument (Parker, 1989; Parker et al., 1979) (∝ = 0.89 for father-child bonding; ∝ = 0.89 for mother-child bonding). Items referred to parents’ behavior in the past six months (e.g., “spoke to me in a warm and friendly voice”). Response options were “usually or always,” “often/a lot,” “not very often,” and “rarely or never.” Responses were summed across items, such that higher scores indicate greater bonding.

#### Parent-child closeness

Two items from the C-SSAGA-A-IV were used to measure father-and mother-child closeness: “how well do you get along with your [father/mother] figure most of the time?” (1 = *poor*, 2 = *fair*, 3 = *good*, 4 = *excellent*), and “how close do you feel to your [father/mother] figure?” (1 = *not at all close*, 2 = *somewhat close*, 3 = *very close*). Responses were standardized and averaged across the two items (Su et al., 2018). The values of Cronbach’s alpha were ∝ = 0.72 for father-child closeness and ∝ = 0.69 for mother-child closeness.

#### Parent-child communication

Father- and mother-child communication were assessed using three items from the C-SSAGA-A-IV, which asked adolescents whether they discussed the news, their friends and activities, and their problems with their parents (∝ = 0.59 for father-child communication; ∝ = 0.56 for mother-child communication). Responses were coded as 0 (“no”) and 1 (“yes”) before calculating a sum score (Su et al., 2018).

#### Parental involvement

Paternal and maternal involvement were assessed using six items from the C-SSAGA-A-IV, which evaluated fathers’ and mothers’ involvement with schoolwork or projects, chores, fun activities, shopping, and making plans (∝ = 0.64 for paternal involvement; ∝ = 0.64 for maternal involvement). Response options were coded as 0 (“no”) and 1 (“yes”) before calculating a sum score (Su et al., 2018).

#### Parental monitoring

Three items from the C-SSAGA-A-IV measured parental monitoring (∝ = 0.73): “my parent figures know about my plans,” “my parent figures have a pretty good idea of my interests, activities, and whereabouts,” and “my parent figures know where I am and who I am with when I am not at home” (1 = *always*, 2 = *usually*, 3 = *sometimes*, 4 = *rarely*). Responses were reverse coded and summed across items (Chassin et al., 1993).

#### Peer drinking

The C-SSAGA-A-IV measured peer drinking with one item: “how many of your best friends use alcohol?” Response options were “none of them” (1), “a few of them” (2), “most of them” (3), and “all of them” (4) (Kaprio et al., 2002).

#### Romantic partner drinking

Drinking by romantic partners was assessed with one item from the C-SSAGA-A-IV: “have you had any boyfriends or girlfriends who used alcohol?” Partner drinking was a dichotomous variable, with responses coded as “no” (0) or “yes” (1).

#### Social competence

Social competence was measured using the six-item social competence scale from the Achenbach Youth Self-Report, which assesses adolescents’ number of group activities, number of friends, frequency of social interactions, and quality of social interactions (Achenbach & Rescorla, 2001; Ivanova et al., 2007; Song et al., 1994).

#### Polygenic risk scores (PRS)

Three genome-wide genotyping arrays were used in COGA Phase IV: data for individuals of European ancestry (EA) were genotyped on the Illumina Human OmniExpress 12V1 array (Illumina, San Diego, CA); data for individuals of African ancestry (AA) were genotyped on the Illumina 2.5M array (Illumina, San Diego, CA); and all remaining samples were genotyped on the Smokescreen genotyping array (BioRealm LLC, Walnut, CA). Samples were imputed to 1000 Genomes using SHAPEIT2 (Delaneau et al., 2013) and Minimac3 (Das et al., 2016) within each array. Variants with non-A/T or C/G alleles, missing rates less than 5%, minor allele frequencies greater than 3%, and Hardy-Weinberg equilibrium *p*-values more than 0.0001 were used for imputation. Imputed single nucleotide polymorphisms (SNPs) with information scores less than 0.30, individual genotype probability scores less than 0.90, a missing rate greater than 25%, or a minor allele frequency less than 1% were excluded, as were SNPs that did not pass Hardy-Weinberg equilibrium (*p* < 1 × 10^−6^). In total, 6,832,792 SNPs passed quality control thresholds and were available for analysis (Lai et al., 2019).

PRS were constructed using PRS-CSx (Ruan et al., 2022). This procedure uses ancestry-specific discovery sample genome-wide association study (GWAS) weights, paired with linkage disequilibrium information from an ancestry-matched external reference panel, to estimate the posterior effect size for each SNP. By integrating GWAS summary statistics from multiple populations, PRS-CSx improves polygenic prediction in non-European samples. Reference panels from the 1000 Genomes Phase III European or African subsamples were used for the EA and AA groups, respectively. For participants of European ancestry, the discovery sample consisted of a meta-analysis of summary statistics from the following GWAS performed in individuals of European ancestry: DSM-IV alcohol dependence from the Psychiatric Genomics Consortium (COGA sample removed; *N* = 45,622) (Walters et al., 2018), AUDIT-P from the UKBiobank (*N* = 141,932) (Sanchez-Roige et al., 2019), and DSM-5 Alcohol Use Disorder from the Million Veteran Program (*N* = 202,004) (Kranzler et al., 2019). For participants of African ancestry, GWAS summary statistics were drawn from the meta-analyzed European ancestry summary statistics described above, in tandem with summary statistics from the following GWAS performed in individuals of African ancestry: DSM-IV alcohol dependence from the Psychiatric Genomics Consortium (COGA sample removed; *N* = 6,280) (Walters et al., 2018) and DSM-5 Alcohol Use Disorder from the Million Veteran Program (*N* = 56,648) (Kranzler et al., 2019).

#### Family history density for AUD

Family history density for AUD was calculated based on log-transformed DSM-5 AUD maximum symptom counts for all first- and second-degree non-descendants, including fathers, mothers, full siblings, grandparents, parental siblings, and half siblings. Maximum AUD symptom counts were weighted according to degree of relatedness (Pandey et al., 2020).

#### Age of alcohol use initiation

Age of alcohol use onset was measured within the C-SSAGA-A-IV: “how old were you the first time you had your very first whole drink?”

#### Heavy episodic drinking onset

Initiation of heavy episodic drinking was assessed using one item from the C-SSAGA-A-IV and from the adult version of the Semi-Structured Assessment for the Genetics of Alcoholism (SSAGA-IV): “did you ever have five or more drinks in 24 hours?” Age of heavy episodic drinking onset was coded as the participant’s age at the first assessment in which they report heavy episodic drinking.

#### AUD diagnosis

AUD diagnoses were evaluated using the C-SSAGA-A-IV and Semi-Structured Assessment for the Genetics of Alcoholism. Individuals aged 15 years or older who met diagnostic criteria for DSM-IV alcohol dependence were coded as affected (0). Drinkers who were assessed through age 23 and did not meet diagnostic criteria at any evaluation were coded as 1, and individuals who did not drink or who were not assessed through age 23 and did not meet criteria for AUD at any evaluation were coded as missing. By removing individuals who were not assessed through age 23, this coding scheme ensures that individuals who have not passed the peak period of vulnerability for AUD are not improperly identified as unaffected (Wang et al., 2013).

#### Covariates

Age at the baseline assessment, sex (0 = male, 1 = female), 10 ancestral principal components, presence of data for maternal AUD, and presence of data for paternal AUD were included as covariates in Cox proportional hazard models and in models involving resistance to AUD. The latter two covariates were included to correct for our earlier observation that missing parental information is often associated with increased risk for externalizing behaviors and substance use disorders in this sample (Salvatore et al., 2015).

### Statistical analysis

#### Preliminary analyses

To construct measures of father-child and mother-child relationship quality, separate one-factor confirmatory factor analyses were conducted using the “cfa” function in the R {lavaan} package (Rosseel, 2012). Parent-child bonding, closeness, communication, and involvement were included as indicators. A Comparative Fit Index (CFI) > 0.90 and Standardized Root Mean Squared Residual (SRMR) < 0.08 were considered as criteria for acceptable model fit (Hu & Bentler, 1999). The “lavPredict” function was then used to extract factor scores for mother-child and father-child relationship quality.

#### Operationalizing realized alcohol resistance

To calculate the alcohol resistance phenotype, two mixed effects Cox proportional hazard models were conducted using the “coxph” function in the R {survival} package (Therneau, 2015). Mixed effects models were used to account for non-independence of data from the same family. Family history density and polygenic risk for AUD were included as predictors, and age at the baseline assessment, sex, 10 ancestral principal components, presence of maternal data for AUD, and presence of paternal data for AUD were included as covariates. The first Cox proportional hazard model evaluated resistance to alcohol use onset; only individuals who had not initiated alcohol use at the baseline assessment (*N* = 1,141) were included. The second Cox proportional hazard model assessed resistance to heavy episodic drinking, and analyses were limited to individuals who did not report heavy episodic drinking at baseline (*N* = 1,350). Individuals who had initiated alcohol use or heavy episodic drinking at baseline were excluded to ensure that predictor variables measured at the baseline assessment temporally preceded the alcohol resistance outcome. Hazard ratios (HRs) were considered statistically significant if the 95% confidence interval (CI) did not include 1.

The resistance phenotypes were derived separately within each ancestral group to avoid population stratification. To assess ancestry, principal components (PCs) were calculated using Eigenstrat (Price et al., 2006) and 1000 Genomes. Each individual was assigned an ancestry classification (EA, AA, or Other) based on the first two PCs. Final participant classification as EA versus AA was then derived from family-based analyses: families were assigned an ancestry according to the majority of individual-based ancestry classifications (Lai et al., 2019).

Deviance residuals, which represent the difference between actual age of onset and expected age of onset based on family history density for AUD and PRS, were then extracted from each model. *Positive values* for the deviance residual indicate that the participant’s age of onset occurred sooner than expected based on their level of genetic risk, denoting *lower levels* of realized alcohol resistance. Conversely, *negative values* indicate that the participant’s age of onset occurred later than expected, denoting *higher levels* of resistance.

#### Univariable associations

First, univariable models were constructed to investigate the influence of adolescent relationships with parents, peers, and romantic partners on resistance to alcohol use onset, initiation of heavy episodic drinking, and AUD diagnosis. Features of the parent-child relationship were measured at the baseline assessment, and measures of adolescent relationships with peers and romantic partners were drawn from the first follow-up assessment. Each predictor was included in a separate model to avoid issues with multicollinearity or suppression effects. For initiation of alcohol use and heavy episodic drinking, resistance was operationalized as the deviance residual. Linear mixed models were tested using the R {plm} package (Croissant & Millo, 2008) to account for non-independence of data from individuals within the same family.

For AUD, age at baseline, sex, ancestry, presence of data for maternal AUD, and presence of data for paternal AUD were included as covariates. Resistance was operationalized as the absence of an AUD diagnosis. Resistance to AUD was operationalized differently from resistance to alcohol use initiation and heavy episodic drinking for two reasons. First, any individual who did not meet criteria for AUD by the end of the study (∼90% of the sample) would be right-censored in a Cox proportional hazard model. The deviance residual, by default, is negative for right-censored observations, which likely would have led to a skewed distribution of deviance residuals with restricted variance. Second, though later initiation of alcohol use may be considered a “healthy” developmental outcome, AUD is associated with clinically significant problems and functional impairment, regardless of the age at diagnosis. Because the definition of realized resistance presupposes high biological risk (Vanyukov et al., 2016), only participants who had a non-zero value on the measure of family history density or were in the top quartile for PRS were included in analyses for resistance to AUD (*N* = 1,725). Probit regression models were conducted using the R {pglm} package (Croissant, 2017), which allows for estimation of panel models with a dichotomous outcome and accounts for non-independence of data from individuals within the same family.

#### Structural equation modeling

**Figure 1.**
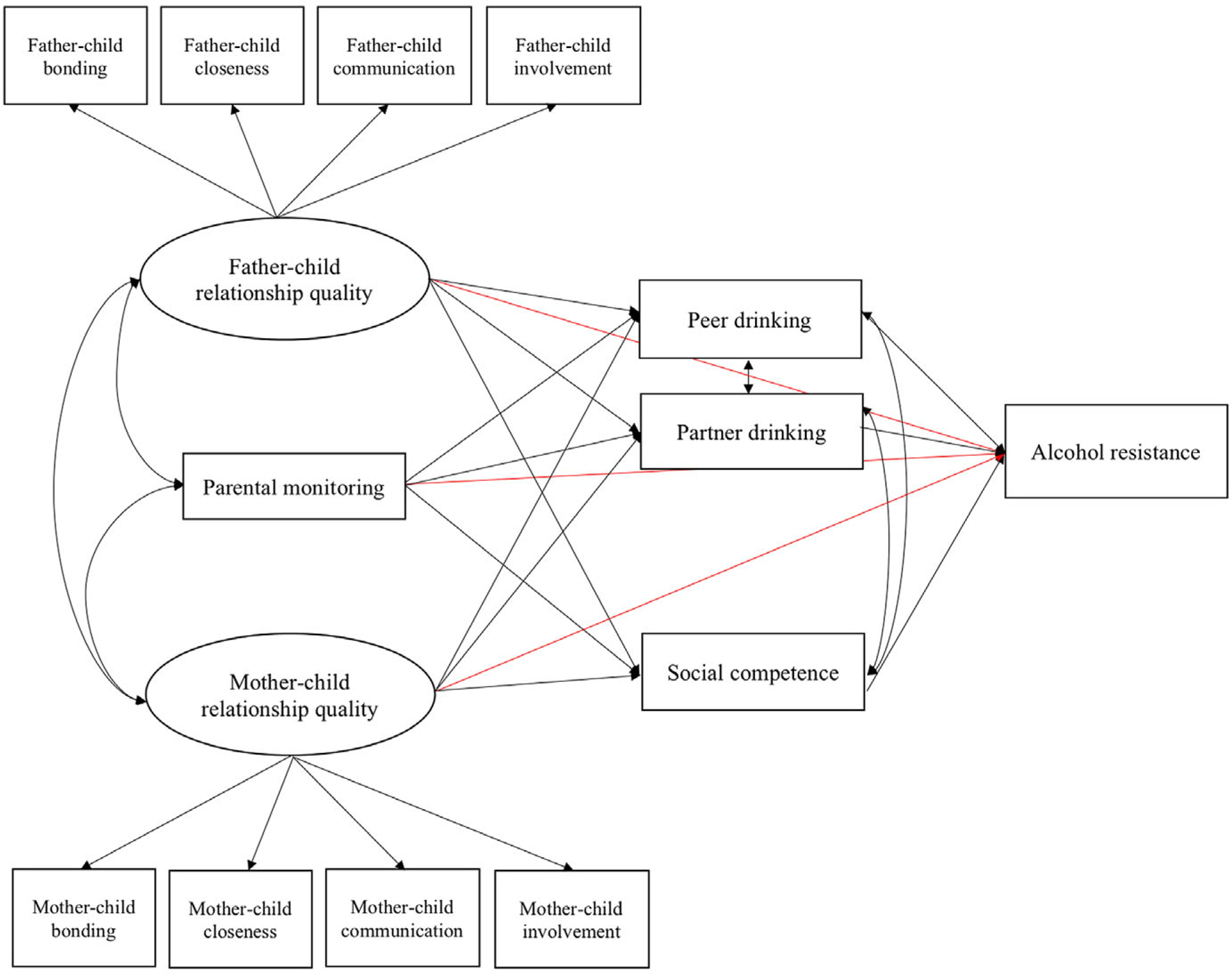
Structural equation model examining the influences of mother-child relationship quality, father-child relationship quality, parental monitoring, peer drinking, partner drinking, and social competence on alcohol resistance. The direct effects of parenting variables on alcohol resistance are shown in red. Variances are not displayed.

Next, consistent with the proposition that familial relationships form the basis for relationships with peers and romantic partners (Sroufe et al., 2005), structural equation models were constructed to evaluate the associations between father-child relationship quality, mother-child relationship quality, and parental monitoring at the baseline assessment and peer drinking, romantic partner drinking, and social competence at the first follow-up assessment. In addition, direct associations of father-child relationship quality, mother-child relationship quality, parental monitoring, peer drinking, romantic partner drinking, and social competence with alcohol resistance were tested (Figure 1). Separate models were constructed for each alcohol resistance outcome using the R {lavaan} package (Rosseel, 2012). Full information maximum likelihood was used to account for missing data.

#### Potential differences in the development of alcohol resistance by ancestry and sex

To test for differences in the pathways to alcohol resistance by participant ancestry, separate models were constructed for individuals of European and African ancestry. Parameter estimates were then constrained to equality across groups, and change in model fit was assessed using the likelihood ratio test. We note that ancestral group and self-reported race/ethnicity are not perfectly correlated but generally correspond to one another (> 96% concordance in the COGA sample). The same procedure was used to test for potential differences in the pathways to alcohol resistance by participant sex.

In view of our preregistered hypotheses, we used *p* < .05 (two-tailed) as the criterion for statistical significance.

## Results

### Preliminary analyses

Descriptive statistics for each of the key study variables are presented in Table 1. One-factor models of parent-child relationship quality were constructed with bonding, closeness, communication, and involvement included as indicators. Confirmatory factor analyses showed acceptable model fit for father-child (χ^2^(2) = 42.71, *p* < .001, CFI = 0.98, SRMR = 0.02) and mother-child (χ^2^(2) = 45.87, CFI = 0.98, SRMR = 0.03) relationship quality.

Next, mixed effects Cox proportional hazard models were conducted to evaluate associations of family history density and polygenic risk for AUD with age of onset for alcohol use and heavy episodic drinking. Age at the baseline assessment, sex, 10 ancestral principal components, presence of maternal data for AUD, and presence of paternal data for AUD were included as covariates, and analyses were conducted separately by ancestry. In these models, family history density and polygenic risk for AUD were not significantly associated with age of alcohol use initiation among EA (family history: HR = 1.12, 95% CI = 0.24, 5.17; polygenic risk: HR = 0.99, 95% CI = 0.46, 2.13) or AA (family history: HR = 1.30, 95% CI = 0.30, 5.61; polygenic risk: HR = 0.92, 95% CI = 0.46, 1.81) individuals. Family history density was associated with earlier onset of heavy episodic drinking among EA individuals (HR = 4.64, 95% CI = 1.10, 19.67) but not AA individuals (HR = 2.35, 95% CI = 0.52, 10.63). Therefore, among EA individuals, a one-unit increase in the family history density score increased the hazard of transition to heavy episodic drinking by a factor of 4.64, on average. Polygenic risk for AUD was not related to age of onset for heavy episodic drinking in either ancestral group (EA individuals: HR = 0.71, 95% CI = 0.33, 1.55; AA individuals: HR = 0.88, 95% CI = 0.39, 2.00). Deviance residuals, which represent the difference between actual age of onset and expected age of onset based on family history density and PRS, were extracted from each model as a measure of realized resistance to alcohol initiation and onset of heavy episodic drinking.

### Univariable associations between adolescent social relationships and alcohol resistance

A series of univariable models were conducted to investigate associations of adolescent relationships with parents, peers, and romantic partners with realized alcohol resistance. Parameter estimates and 95% CIs are presented in Table 2. It should be noted that positive values for the deviance residual indicate that the participant’s age of onset occurred sooner than expected based on their level of genetic risk. Therefore, positive beta estimates indicate that the predictor of interest is related to *lower* levels of realized alcohol resistance, and negative beta estimates indicate that the predictor of interest is related to *higher* levels of resistance. Resistance to AUD, however, was not operationalized using a deviance residual, and parameter estimates may be interpreted in the usual way (i.e., a positive beta estimate indicates that the predictor is related to higher levels of resistance to AUD).

As shown in Table 2, greater father-child relationship quality promoted resistance to alcohol initiation. Conversely, peer and romantic partner drinking were associated with lower levels of resistance to alcohol initiation, and higher levels of peer drinking were also associated with lower resistance to heavy episodic drinking. The remaining associations were not statistically significant, and the observed effect sizes were small. Only the magnitude of the association between romantic partner drinking and resistance to AUD exceeded 0.20; specifically, for individuals who reported that their romantic partner had used alcohol, their z-score for resistance to AUD decreased by 0.31 compared to individuals whose romantic partners had never used alcohol. However, this relationship was not statistically significant.

### Structural equation modeling

Three models were specified to, firstly, evaluate the degree to which family relationships form the basis for relationships with peers and romantic partners and, secondly, to test effects of father- and mother-child relationship quality, parental monitoring, peer drinking, romantic partner drinking, and social competence on resistance to alcohol initiation, onset of heavy episodic drinking, and AUD. Standardized factor loadings, path coefficients, and correlation coefficients for each model are reported in Table 3.

#### Associations of parenting variables with features of peer and romantic relationships

Models of resistance to alcohol initiation, onset of heavy episodic drinking, and AUD included different analytic subsamples: analyses of resistance to alcohol initiation included 1,141 individuals who had not initiated alcohol use at the baseline assessment, analyses of resistance to heavy episodic drinking included 1,350 individuals who did not report heavy episodic drinking at baseline, and analyses of resistance to AUD included participants who had a non-zero value on the measure of family history density or were in the top quartile for PRS (*N* = 1,725). As a result, each model yielded similar, but slightly varying patterns of associations between features of the parent-child relationship and features of peer and romantic relationships in adolescence.

##### Associations with peer drinking.

Across all three models, father-child relationship quality was not significantly associated with peer drinking. Effects of father-child relationship quality on peer drinking were consistently negative and small in magnitude $\left(\left|\hat{\beta }\right|=0.03-0.07\right)$. Greater mother-child relationship quality was associated with lower levels of peer drinking in models of resistance to alcohol initiation and heavy episodic drinking, though this association was non-significant and smaller in magnitude within the model of resistance to AUD. Similarly, higher levels of parental monitoring were associated with lower levels of peer drinking in models of resistance to heavy episodic drinking and AUD, but this association was non-significant and smaller in magnitude within the model of resistance to alcohol initiation.

##### Associations with romantic partner drinking.

Across models, father-child relationship quality was not significantly associated with partner drinking. Effects of father-child relationship quality on partner drinking were consistently negative and small in magnitude $\left(\left|\hat{\beta }\right|=0.10-0.14\right)$. Adolescents who reported higher quality relationships with their mothers were less likely to indicate that their romantic partner consumed alcohol across all three models tested $\left(\left|\hat{\beta }\right|=0.04-0.16\right)$, though the relationship was statistically significant in the model of resistance to AUD only. Parental monitoring was not significantly related to romantic partner drinking across models, though the direction of effect was consistently negative $\left(\left|\hat{\beta }\right|=0.04-0.07\right)$.

##### Associations with social competence.

Father-child relationship quality was not significantly related to social competence and showed varying directions of effect across models. By contrast, higher mother-child relationship quality promoted greater social competence in models of resistance to heavy episodic drinking and AUD, though the association was non-significant and smaller in magnitude within the model of resistance to alcohol initiation. Moreover, higher levels of parental monitoring were related to greater social competence in models of resistance to alcohol initiation and heavy episodic drinking, but the association was non-significant and smaller in magnitude within the model of resistance to AUD.

#### Resistance to alcohol initiation

Greater father-child relationship quality promoted higher resistance to alcohol initiation. Mother-child relationship quality was not significantly associated with resistance to alcohol initiation, though the effect size was non-trivial $\left(\left|\hat{\beta }\right|=0.16\right)$. Effects of parental monitoring, peer drinking, partner drinking, and social competence on resistance were not statistically significant and were extremely small in magnitude $\left(\left|\hat{\beta }\right|=0.00-0.08\right)$.

#### Resistance to heavy episodic drinking

Greater social competence in adolescence was related to lower levels of resistance to heavy episodic drinking. Effects of father-child relationship quality, mother-child relationship quality, parental monitoring, peer drinking, and romantic partner drinking were non-significant and small in magnitude $\left(\left|\hat{\beta }\right|=0.02-0.07\right)$.

#### Resistance to AUD

No statistically significant predictors of resistance to AUD were identified, and effects were small in magnitude $\left(\left|\hat{\beta }\right|=0.00-0.09\right)$.

#### Potential differences in the development of alcohol resistance by ancestry and sex

To test for differences in the pathways to alcohol resistance by participant ancestry, separate models were constructed for participants of European and African ancestries. When comparing a freely estimated model to a model with all paths constrained to equality, there was not a significant change in model fit for models of resistance to alcohol initiation (χ^2^(15) = 10.91, *p* = .759) and heavy episodic drinking (χ^2^(15) = 15.21, *p* = .436). However, there was a statistically significant decrement in model fit when regression coefficients were constrained to be equal within the model of resistance to AUD (χ^2^(15) = 26.21, *p* = .036). Parameter estimates for the freely estimated model are shown in Table 4. To provide further insight into differences in the development of resistance to AUD based on ancestry, each regression path was individually constrained to equality. There was a significant decrease in model fit when the association between mother-child relationship quality and social competence was constrained to be equal across EA and AA groups, χ^2^(1) = 5.46, *p* = .019. Likelihood ratio tests were not statistically significant for the remaining paths, χ^2^(1) = 0.00– 3.45, *p*s = .063–.950.

Next, separate models were specified for males and females, and regression coefficients were constrained to be equal across groups. There was not a significant change in model fit across models of resistance to alcohol initiation (χ^2^(15) = 7.23, *p* = .951), heavy episodic drinking (χ^2^(15) = 13.30, *p* = .579), and AUD (χ^2^(15) = 7.85, *p* = .930), suggesting that pathways to alcohol resistance were similar for male and female participants.

## Discussion

In this study, we used an organizational-developmental perspective (Sroufe et al., 2005) to examine the contributions of adolescent social relationships with parents, peers, and romantic partners to realized alcohol resistance across a series of key drinking milestones – alcohol initiation, the onset of heavy episodic drinking, and diagnosis with alcohol dependence. We focus on four key take-aways from the results of this preregistered study.

First, using conventional thresholds for statistical significance (*p* < .05), we found mixed support for the hypothesis that features of the parent-child relationship would be associated with features of subsequent peer and romantic partner relationships. Father-child relationship quality was not significantly associated with peer drinking, romantic partner drinking, and social competence. Higher mother-child relationship quality was prospectively associated with lower levels of peer drinking in the models of resistance to alcohol initiation and heavy episodic drinking, lower likelihood of romantic partner drinking in the model of resistance to AUD, and higher levels of social competence in the models of resistance to heavy episodic drinking and AUD. Finally, parental monitoring was related to lower levels of peer drinking in the models of resistance to heavy episodic drinking and AUD, and higher levels of social competence in the models of resistance to alcohol initiation and heavy episodic drinking.

It is worth noting that, in instances where a predictor was statistically significant in one of the resistance models but not others (e.g., mother-child relationship quality was associated with lower peer drinking in models of resistance to alcohol initiation and heavy episodic drinking, but not in the model of resistance to AUD), the non-significant effects were in the same direction but less precisely known (as indicated by wider confidence intervals). Thus, taken as a whole, the pattern of results observed here is somewhat consistent with prior theory and evidence regarding the familial antecedents of social competence (Laible & Carlo, 2004) and affiliations with antisocial peers (Dishion et al., 1991). Our results also underscore the need to measure and examine the separate influences of mothers’ and fathers’ parenting, as mother-child relationship quality appeared to have more consistent associations with features of adolescent peer and romantic relationships compared to father-child relationship quality.

Second, we found little evidence to support the hypothesis that features of adolescent social relationships with parents, peers, and romantic partners would promote resistance to alcohol initiation, heavy episodic drinking, and the development of disorder. There were several exceptions: higher father-child relationship quality was associated with higher resistance to alcohol initiation, peer and romantic partner drinking were associated with lower resistance to alcohol initiation (univariable model only), more peer drinking was associated with lower resistance to heavy episodic drinking (univariable model only), and adolescent social competence was associated with *lower resistance* to heavy episodic drinking. As noted by others (Bell et al., 2020), research on the impact of parenting on adolescent substance use outcomes has historically focused on mothers or used non-specific language when referring to parents. In an illustration of this, only two father-specific effects for parent-child relationship quality and alcohol initiation were identified as part of a recent meta-analysis of the relationship between parenting factors and adolescent alcohol misuse (Yap et al., 2017). Thus, our finding that father-child relationship quality influences resistance to alcohol initiation adds to an emerging area of research on the developmental significance of fathers for adolescent alcohol outcomes and merits replication in other samples.

Although the association between adolescent social competence and *lower resistance* to heavy episodic drinking was unexpected, we speculate that this effect may reflect the benefits and liabilities associated with social competence in this period of the life span. On the one hand, the ability to establish high quality relationships with friends and successfully navigate the larger peer group is a hallmark of adolescent social functioning (Brown, 2004). Yet, this level of social integration can also render one vulnerable to risky behaviors, such as alcohol use, that often take place in group contexts. Indeed, given that adolescent drinking is associated with higher quality relationships with friends and largely takes places in social contexts (Hoel et al., 2004; Hughes et al., 1992), some developmentalists have noted that adolescent alcohol use can be considered a marker of social integration and adaptation (Jessor, 1991; Vaillant, 1995).

Third, we identified very few differences in pathways to alcohol resistance based on participant sex and ancestry. For models of resistance to alcohol initiation and heavy episodic drinking, regression coefficients could be constrained to equality without a substantial change in model fit. For resistance to AUD, only the association between mother-child relationship quality and social competence was significantly different among European and African ancestry individuals. Specifically, higher mother-child relationship quality promoted greater social competence among individuals of European, but not African, ancestry.

Finally, given the relative novelty of the resistance framework, it is worth commenting more broadly on the opportunities and challenges of this approach as it relates to alcohol outcomes. Vanyukov et al. (2016) have argued that resistance factors are not merely risk factors with the opposite sign. Just as trauma exposure may elevate risk for alcohol misuse, but a lack of trauma exposure is not necessarily protective, resistance factors likely encompass a number of environmental variables whose presence enhances resistance but whose absence may be considered neutral. As an illustrative example, consider the Icelandic Prevention Model for adolescent substance use prevention. One of the most highly utilized components of the prevention program was the “parental prowl,” in which parents joined together to walk around their neighborhood and monitor youth during Friday and Saturday evenings. Though the *lack* of a community monitoring approach is not necessarily risky, the “parental prowl” enhanced neighborhood social capital and contributed to a substantial decline in adolescent substance use following the implementation of the Icelandic Prevention Model (Sigfúsdóttir et al., 2009). Such findings also bolster Vanyukov and colleagues’ (2016) argument that resistance factors may have stronger translational impact than risk factors, given their potentially broader application and direct relationship with healthy outcomes. Yet, the largely null effects observed across the candidate resistance factors examined here underscores the need for further theoretical articulation of the factors that contribute to realized resistance. Answering these questions is critical for understanding the factors that can promote healthy outcomes among those at the greatest genetic risk for AUD.

### Limitations

Our results should be interpreted within the context of the following limitations. First, COGA is a high-risk sample with most participants from extended families enriched for AUDs. Findings may not be generalizable to other populations or samples ascertained with different risk profiles. Second, individuals who drank earlier than their first assessment were coded as missing from the analyses of resistance to alcohol initiation to avoid a situation where the parenting, peer, and romantic partner predictor variables were measured after the age at initiation. As a consequence, very early drinkers were removed from the analysis, and our results are not informative about resistance to alcohol initiation in especially young age groups. Third, although the identification of potential resistance factors for the present study was grounded in an organizational-developmental perspective, it is also acknowledged they were selected from a study that (like many) was originally conceived within a risk framework (Bucholz et al., 2017). Fourth, we recognize that our measure of parental monitoring reflects not only parental behavior, but also adolescents’ disclosure of their activities and whereabouts (Stattin & Kerr, 2000).

Fifth, we relied on genome-wide polygenic scores and family history measures to calculate the measures of realized resistance. Although this approach was chosen to align with Vanyukov et al.’s (2016) model, we recognize that polygenic scores and family history measures represent imprecise measures of genetic risk, and also capture some environmental risk (Selzam et al., 2019). Further, polygenic scores were not statistically significantly associated with age at initiation of alcohol use and heavy episodic drinking in the present study, which limits the utility of deviance residuals and may contribute to the pattern of largely null effects. As GWAS discovery sample sizes continue to increase, the predictive accuracy of polygenic risk scores (Dudbridge, 2013) and utility of deviance residuals should correspondingly improve, enabling future studies of realized resistance. Sixth, polygenic scores for participants of African ancestry were based on summary statistics from both European ancestry and African ancestry discovery samples (with appropriate adjustments to the European ancestry summary statistics). This method was selected to improve polygenic prediction in individuals of African ancestry, as existing GWAS of alcohol-related outcomes in African ancestry individuals have very small sample sizes relative to GWAS in European ancestry individuals. Continued efforts to improve the representation of diverse populations in genetic research may facilitate the use of ancestrally matched discovery samples and further strengthen polygenic prediction in African ancestry individuals.

Seventh, genetic influences on features of the environment, including parenting, were not addressed in the present work. It is certainly plausible that familial or polygenic loading for AUD is correlated with parent-child relationship quality and parental monitoring (see Kendler & Baker, 2007 for a review of genetic influences on the environment). However, within the context of the current study, we anticipate that the presence of gene-environment correlation would attenuate, rather than strengthen, the magnitude of associations. In the presence of gene-environment correlation, parenting behaviors should contribute to greater correspondence between the adolescent’s genetic risk and alcohol-related outcome, rather than facilitating resistance to genetic or familial risk for AUD. Eighth, we focused on realized resistance in this study. Whether the candidate resistance factors examined here are associated with other dimensions of resistance, such as resistance to relapse following recovery, remains to be seen. Finally, although many of our hypothesized effects were not statistically significant, we caution that these null effects do not imply that relationships with parents, peers, and romantic partners do not contribute to alcohol resistance.

## Conclusions

Historically, efforts to understand the etiology of health and disorder have focused on identifying risk and protective factors. A resistance framework (Vanyukov et al., 2016) represents a shift in that paradigm to instead ask what promotes a healthy outcome among individuals with a high liability to disorder. In the present study, results were largely inconsistent with our hypotheses that parenting, peer, and romantic partner factors would contribute to resistance to key drinking milestones, with the exception that father-child relationship quality promoted resistance to alcohol initiation. This pattern of null effects illustrates how little is known about resistance processes among those at high genetic risk for AUD. Identifying variables that increase risk, then seeking to reduce or eliminate those risk factors, has provided only partial insight into how individuals remain healthy despite high biological risk for disorder. To further our understanding of healthy developmental outcomes, searching for and bolstering resistance-enhancing factors is an important next step.

## Figures and Tables

**Table 1.** Descriptive statistics for key study variables

Variable	Subsample 1: Resistance to alcohol initiation (*N* = 1141)			Subsample 2: Resistance to HED (*N* = 1350)			Subsample 3: Resistance to AUD (*N* = 1725)		
	*N*	*M/*%	*SD*	*N*	*M/*%	*SD*	*N*	*M/*%	*SD*
Sex (% female)	1141	49.95%	-	1350	49.95%	-	1725	49.95%	-
Ancestry (% AA)	1141	36.52%	-	1350	36.52%	-	1725	36.52%	-
Age at alcohol initiation	1141	16.01	2.57	1345	15.56	2.69	1479	15.20	2.73
Age at HED onset	1141	19.13	3.34	1350	19.17	3.28	1725	18.99	3.08
AUD diagnosis	1141	8.62%	-	1350	9.69%	-	1725	10.61%	-
Family history	1141	0.39	0.17	1350	0.40	0.17	1725	0.42	0.18
PRS	1121	2.11	1.05	1331	2.12	1.04	1698	2.16	1.01
F-C bonding	740	39.42	6.84	846	39.04	7.01	1086	38.81	7.06
F-C closeness	958	0.05	0.85	1123	0.01	0.87	1424	−0.02	0.89
F-C communication	958	2.03	1.03	1123	2.00	1.04	1424	1.97	1.04
F-C involvement	958	3.82	1.56	1123	3.71	1.58	1424	3.67	1.62
M-C bonding	804	41.35	6.01	920	40.99	6.30	1185	40.75	6.56
M-C closeness	1133	0.07	0.77	1339	−0.01	0.85	1707	−0.02	0.89
M-C communication	1133	2.39	0.86	1339	2.33	0.89	1707	2.31	0.91
M-C involvement	1133	4.61	1.30	1339	4.52	1.36	1707	4.48	1.38
Parental monitoring	1141	7.32	1.85	1350	7.18	1.92	1725	7.07	2.00
Peer drinking	1061	1.45	0.68	1147	1.51	0.73	1724	1.56	0.77
Partner drinking	556	2.17	1.82	623	2.25	1.85	859	2.32	1.87
Social competence	550	8.32	2.38	594	8.30	2.39	1340	8.35	2.38

*Notes*. Subsamples for analyses of resistance to alcohol initiation, heavy episodic drinking, and AUD differed in size because participants who endorsed the outcome of interest (initiation of alcohol use, initiation of heavy episodic drinking, and AUD diagnosis, respectively) at the baseline assessment were excluded. Analyses of resistance to AUD were further limited to participants who had a non-zero family history density score or were in the top quartile for PRS. Abbreviations. AA = African ancestry; HED = heavy episodic drinking; AUD = alcohol use disorder; PRS = polygenic risk score; F-C = father-child; M-C = mother-child.

**Table 2.** Univariable associations between predictor variables and resistance to alcohol initiation, heavy episodic drinking, and alcohol use disorder

*Variable*	Resistance to alcohol initiation	Resistance to HED	Resistance to AUD
	$\hat{\beta }$ [95% CI]	$\hat{\beta }$[95% CI]	$\hat{\beta }$[95% CI]
F-C relationship quality	**−0.06 [−0.12, 0.00]**	−0.02 [−0.07, 0.04]	0.07 [−0.09, 0.22]
M-C relationship quality	0.03 [−0.03, 0.09	0.01 [−0.04, 0.06]	0.09 [−0.04, 0.22]
Parental monitoring	−0.01 [−0.04, 0.02]	−0.01 [−0.03, 0.02]	0.04 [−0.03, 0.10]
Peer drinking	**0.13 [0.05, 0.20]**	**0.15 [0.09, 0.22]**	−0.11 [−0.32, 0.09]
Partner drinking	**0.27 [0.10, 0.43]**	0.15 [0.00, 0.30]	−0.31 [−0.77, 0.14]
Social competence	0.01 [−0.03, 0.04]	0.01 [−0.02, 0.04]	0.07 [−0.03, 0.17]

*Notes*. Statistically significant associations are shown in bold font. Abbreviations. F-C = father-child; M-C = mother-child; HED = heavy episodic drinking; AUD = alcohol use disorder.

**Table 3.** Parameter estimates for models of realized alcohol resistance

*Latent variable*	*Manifest variable*	Resistance to alcohol initiation	Resistance to HED	Resistance to AUD
		*λ* [95% CI]	*λ* [95% CI]	*λ* [95% CI]
F-C rel. quality	F-C bonding	**0.82 [0.76, 0.88]**	**0.80 [0.75, 0.85]**	**0.75 [0.70, 0.79]**
F-C rel. quality	F-C closeness	**0.70 [0.64, 0.76]**	**0.66 [0.61, 0.72]**	**0.71 [0.67, 0.75]**
F-C rel. quality	F-C communication	**0.60 [0.52, 0.67]**	**0.62 [0.56, 0.68]**	**0.62 [0.57, 0.67]**
F-C rel. quality	F-C involvement	**0.62 [0.55, 0.69]**	**0.67 [0.62, 0.73]**	**0.64 [0.59, 0.68]**
M-C rel. quality	M-C bonding	**0.70 [0.62, 0.78]**	**0.74 [0.68, 0.80]**	**0.66 [0.61, 0.71]**
M-C rel. quality	M-C closeness	**0.64 [0.56, 0.72]**	**0.66 [0.60, 0.71]**	**0.67 [0.63, 0.72]**
M-C rel. quality	M-C communication	**0.45 [0.36, 0.54]**	**0.53 [0.46, 0.60]**	**0.57 [0.52, 0.62]**
M-C rel. quality	M-C involvement	**0.52 [0.44, 0.61]**	**0.59 [0.53, 0.66]**	**0.56 [0.51, 0.61]**
*Predictor*	*Outcome*	$\hat{\beta }$[95% CI]	$\hat{\beta }$[95% CI]	$\hat{\beta }$[95% CI]
F-C rel. quality	Peer drinking	−0.03 [−0.19, 0.12]	−0.07 [−0.20, 0.06]	−0.05 [−0.14, 0.04]
M-C rel. quality	Peer drinking	**−0.17 [−0.33, −0.01]**	**−0.19 [−0.32, −0.06]**	−0.06 [−0.15, 0.04]
Parental monitoring	Peer drinking	−0.08 [−0.19, 0.03]	**−0.10 [−0.19, −0.01]**	**−0.20 [−0.27, −0.13]**
F-C rel. quality	Partner drinking	−0.13 [−0.37, 0.11]	−0.14 [−0.31, 0.04]	−0.10 [−0.23, 0.03]
M-C rel. quality	Partner drinking	−0.04 [−0.26, 0.19]	−0.16 [−0.33, 0.01]	**−0.15 [−0.29, −0.02]**
Parental monitoring	Partner drinking	−0.07 [−0.26, 0.11]	−0.04 [−0.17, 0.09]	−0.06 [−0.15, 0.03]
F-C rel. quality	Social competence	−0.10 [−0.27, 0.07]	−0.11 [−0.24, 0.03]	0.09 [−0.02, 0.19]
M-C rel. quality	Social competence	0.07 [−0.10, 0.25]	**0.23 [0.09, 0.38]**	**0.15 [0.04, 0.26]**
Parental monitoring	Social competence	**0.24 [0.13, 0.36]**	**0.15 [0.05, 0.25]**	0.07 [−0.01, 0.15]
F-C rel. quality	Alcohol resistance	**−0.19 [−0.35, −0.03]**	−0.04 [−0.17, 0.10]	0.00 [−0.11, 0.10]
M-C rel. quality	Alcohol resistance	0.16 [−0.01, 0.33]	0.03 [−0.11, 0.18]	0.09 [−0.03, 0.20]
Parental monitoring	Alcohol resistance	−0.08 [−0.20, 0.03]	−0.07 [−0.17, 0.03]	−0.03 [−0.11, 0.05]
Peer drinking	Alcohol resistance	−0.01 [−0.13, 0.10]	0.02 [−0.08, 0.11]	−0.02 [−0.10, 0.05]
Partner drinking	Alcohol resistance	0.00 [−0.19, 0.18]	−0.05 [−0.19, 0.10]	0.03 [−0.07, 0.12]
Social competence	Alcohol resistance	−0.04 [−0.14, 0.06]	**0.10 [0.01, 0.20]**	0.00 [−0.07, 0.07]
*Variable X*	*Variable Y*	*Corr.* [95% CI]	*Corr.* [95% CI]	*Corr.* [95% CI]
F-C rel. quality	M-C rel. quality	**0.63 [0.53, 0.72]**	**0.62 [0.55, 0.70]**	**0.58 [0.51, 0.64]**
F-C rel. quality	Parental monitoring	**0.52 [0.44, 0.60]**	**0.50 [0.43, 0.57]**	**0.44 [0.38, 0.49]**
M-C rel. quality	Parental monitoring	**0.50 [0.41, 0.58]**	**0.54 [0.48, 0.61]**	**0.53 [0.48, 0.58]**
Peer drinking	Social competence	0.04 [−0.05, 0.13]	0.06 [−0.02, 0.14]	0.06 [0.00, 0.12]
Peer drinking	Partner drinking	**0.37 [0.25, 0.48]**	**0.37 [0.29, 0.45]**	**0.41 [0.36, 0.47]**
Partner drinking	Social competence	0.04 [−0.13, 0.21]	0.07 [−0.06, 0.20]	0.04 [−0.05, 0.13]

*Notes.* Statistically significant associations are shown in bold font. *Abbreviations.* F-C = father-child; M-C = mother-child; HED = heavy episodic drinking; AUD = alcohol use disorder.

**Table 4.** Parameter estimates for ancestry-stratified model of resistance to alcohol use disorder

*Latent variable*	*Manifest variable*	European ancestry	African ancestry
		*λ* [95% CI]	*λ* [95% CI]
F-C rel. quality	F-C bonding	**0.83 [0.76, 0.89]**	**0.55 [0.41, 0.70]**
F-C rel. quality	F-C closeness	**0.77 [0.70, 0.85]**	**0.85 [0.76, 0.93]**
F-C rel. quality	F-C communication	**0.63 [0.54, 0.73]**	**0.74 [0.64, 0.85]**
F-C rel. quality	F-C involvement	**0.64 [0.55, 0.73]**	**0.72 [0.61, 0.83]**
M-C rel. quality	M-C bonding	**0.83 [0.76, 0.90]**	**0.64 [0.50, 0.77]**
M-C rel. quality	M-C closeness	**0.77 [0.70, 0.85]**	**0.75 [0.63, 0.86]**
M-C rel. quality	M-C communication	**0.61 [0.51, 0.71]**	**0.64 [0.51, 0.78]**
M-C rel. quality	M-C involvement	**0.57 [0.47, 0.67]**	**0.66 [0.52, 0.79]**
*Predictor*	*Outcome*	$\hat{\beta }$[95% CI]	$\hat{\beta }$[95% CI]
F-C rel. quality	Peer drinking	−0.14 [−0.29, 0.01]	−0.03 [−0.29, 0.22]
M-C rel. quality	Peer drinking	−0.04 [−0.21, 0.12]	0.03 [−0.25, 0.30]
Parental monitoring	Peer drinking	**−0.26 [−0.41, −0.11]**	−0.07 [−0.28, 0.14]
F-C rel. quality	Partner drinking	**−0.20 [−0.35, −0.05]**	−0.13 [−0.38, 0.12]
M-C rel. quality	Partner drinking	−0.01 [−0.18, 0.16]	−0.13 [−0.39, 0.14]
Parental monitoring	Partner drinking	**−0.16 [−0.31, 0.00]**	0.07 [−0.14, 0.27]
F-C rel. quality	Social competence	−0.01 [−0.16, 0.14]	0.16 [−0.09, 0.41]
M-C rel. quality	Social competence	**0.40 [0.25, 0.55]**	−0.07 [−0.34, 0.20]
Parental monitoring	Social competence	0.08 [−0.06, 0.23]	0.12 [−0.09, 0.33]
F-C rel. quality	Alcohol resistance	−0.07 [−0.22, 0.09]	−0.12 [−0.38, 0.13]
M-C rel. quality	Alcohol resistance	0.10 [−0.08, 0.28]	−0.01 [−0.29, 0.26]
Parental monitoring	Alcohol resistance	−0.01 [−0.16, 0.15]	0.14 [−0.07, 0.35]
Peer drinking	Alcohol resistance	−0.15 [−0.31, 0.01]	0.07 [−0.18, 0.32]
Partner drinking	Alcohol resistance	−0.06 [−0.22, 0.10]	−0.17 [−0.42, 0.08]
Social competence	Alcohol resistance	**0.15 [0.01, 0.30]**	−0.03 [−0.22, 0.15]
*Variable X*	*Variable Y*	*Corr.* [95% CI]	*Corr.* [95% CI]
F-C rel. quality	M-C rel. quality	**0.33 [0.19, 0.48]**	**0.53 [0.35, 0.70]**
F-C rel. quality	Parental monitoring	**0.36 [0.23, 0.49]**	**0.38 [0.21, 0.55]**
M-C rel. quality	Parental monitoring	**0.49 [0.38, 0.61]**	**0.44 [0.27, 0.61]**
Peer drinking	Social competence	−0.07 [−0.21, 0.06]	−0.11 [−0.29, 0.07]
Peer drinking	Partner drinking	**0.54 [0.44, 0.63]**	**0.68 [0.58, 0.78]**
Partner drinking	Social competence	0.06 [−0.08, 0.19]	0.00 [−0.18, 0.18]

*Notes*. Statistically significant associations are shown in bold font. Abbreviations. F-C = father-child; M-C = mother-child.
